# Ortho-Endo Management of Traumatically Intruded Immature Incisors: A Case Report of Novel Interventional Approach With 20 Months Follow-Up

**DOI:** 10.7759/cureus.54498

**Published:** 2024-02-19

**Authors:** Raichel M Geevarghese, Ramanandvignesh Pandiyan, Sapna Singla, Manjit Talwar, Gurvanit Lehl

**Affiliations:** 1 Pedodontics and Preventive Dentistry, KIMS Health Medical Center, Qatar, QAT; 2 Pedodontics and Preventive Dentistry, Government Medical College and Hospital, Chandigarh, Chandigarh, IND; 3 Orthodontics and Dentofacial Orthopaedics, Government Medical College and Hospital, Chandigarh, Chandigarh, IND

**Keywords:** injectable platelet-rich fibrin, regenerative endodontics therapy, traumatic dental injury, orthodontic management, intrusion

## Abstract

The case report describes the successful interdisciplinary management of a nine-year-old girl who suffered from traumatic intrusion and rotation of her immature teeth with 12 mm apical displacement of teeth 11 and 21 and 10 mm of teeth 12 and 22. It emphasizes the importance of a comprehensive and contemporary approach, which includes orthodontic intervention and regenerative endodontic procedures (REP). The report follows the CARE (case report) guidelines. The patient was initially observed for four weeks to see if there was potential for spontaneous re-eruption. After eight weeks of orthodontic intervention, the rotation was corrected. However, negative pulp sensitivity and external inflammatory (infection-related) resorption (EIR) occurred, which led to REP using injectable platelet-rich fibrin (i-PRF). Follow-ups over 20 months showed the favorable outcomes of the treatment. A long-term perspective is essential in understanding the outcomes and potential complications associated with traumatic dental injuries (TDI) in children. The case report highlights the importance of collaboration between orthodontists and pediatric dentists, among other specialists. It provides valuable insights into the complexities of managing TDI in children and highlights contemporary regenerative strategies as viable solutions.

## Introduction

In children aged 6-12 years, traumatic injuries to immature permanent teeth are common, posing challenges to their physiological development and causing concerns for physical, psychological, and aesthetic well-being. Among traumatic dental injuries (TDI), intrusive luxation is severe, impacting periodontal fibers, neurovascular bundle, cementum, and alveolar bone, with a prevalence of only 0.3%-1.9% [1-3]. Treatment approaches vary, encompassing spontaneous re-eruption and interdisciplinary methods such as endodontic, orthodontic, and surgical interventions [4]. Despite diverse views on management, prioritizing minimal complications is crucial. This case report aims to delineate the interdisciplinary management of traumatically intruded and rotated incisors with an immature apex, emphasizing the need for individualized approaches.

## Case presentation

A nine-year-old girl reported, 24 hours following a playground accident, at our dental center with traumatic upper front tooth displacement. Clinical examination revealed gingival inflammation and laceration. Intraoral periapical radiograph revealed 12 mm apical displacement of 11 and 21 and 10 mm of 12 and 22 with open apices and uncomplicated fracture of 11 and 21. After a week of prescribed analgesics and 0.2% chlorhexidine gluconate rinse, reduced inflammation and slight visibility of 12 and 22 were noted. The case report underscores the interdisciplinary approach needed for traumatic dental injuries in children, emphasizing the importance of careful management in restoring function and aesthetics. Informed consent was taken from the guardian.

**Figure 1:**
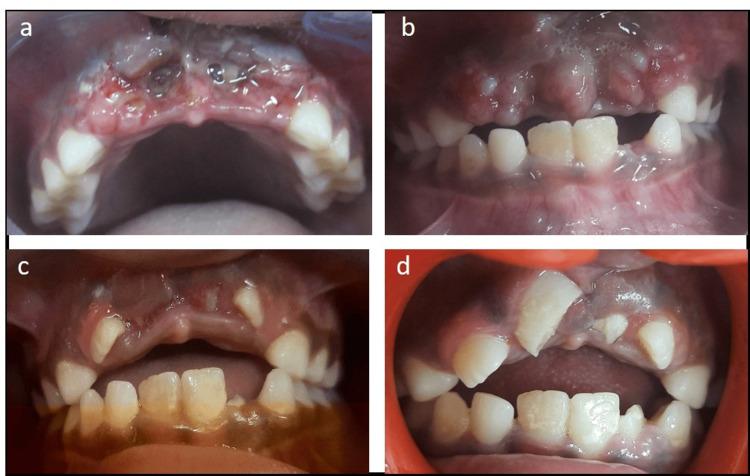
a) Gingival laceration and intruded incisors, b) healing after one week, c) spontaneous eruption of 12 and 22 after four weeks, and d) spontaneous eruption of 11 and 21 after four months.

Due to severe intrusive luxation with immature teeth, the patient was allowed for spontaneous repositioning for four weeks according to the 2020 International Association of Dental Traumatology guidelines (IADT) [5]. Clinical examination at this juncture revealed a continued improvement in the visibility of 12 and 22 in the oral cavity, indicating their normal eruption. However, 11 and 21 remained invisible. In light of this, a decision was made to refrain from intervention at this stage, opting instead for continued observation. The patient was scheduled for subsequent monthly evaluations to monitor the ongoing eruption progress of the 11 and 21. After four months post-injury, only a 4 mm incisal edge of 11 and a mere 1 mm of the incisal angle of 21 were visible in the oral cavity. In response to this limited visibility, a decision was made to initiate orthodontic repositioning of 11 and 21 (Figure 1). Alternative treatment options, such as surgical extrusion, would not be desirable for immature teeth [2,5]. A pulp sensibility test (heat test with gutta-percha) was conducted four months post injury, revealing no response in 11 and 21, while 12 and 22 showed a positive response. Considering the lack of signs, such as infected pulp or external inflammatory (infection-related) resorption (EIR), and aiming to facilitate spontaneous revascularization of the pulp, planned endodontic management for 11 and 21 was deferred until considerable tooth exposure was achieved through orthodontic repositioning.

**Figure 2:**
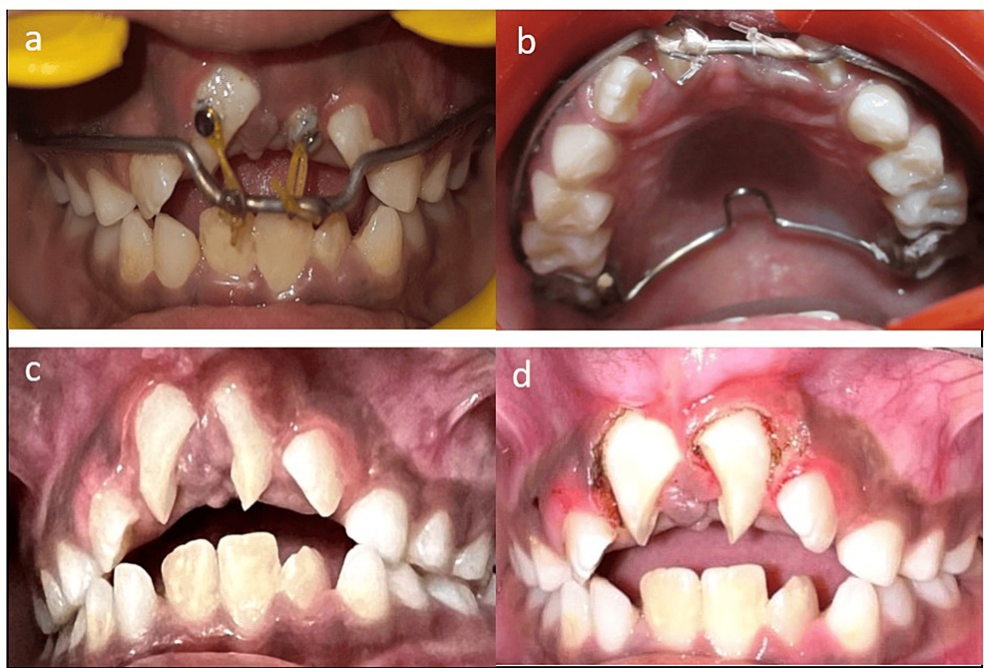
a, b) Orthodontic traction started c) after eight weeks of elastic traction and d) gingivectomy performed for bonding brackets.

To facilitate orthodontic repositioning, an intraoral appliance was custom-made, comprising a trans-palatal arch crafted from a 0.036" stainless steel wire, which was soldered to molar bands to provide anchorage. Additionally, another wire of identical dimensions was adapted to the buccal surfaces and extended onto the labial portion. This wire was strategically bent downward to offer anchorage for the downward traction of the incisors. Buttons were bonded onto the visible labial surface of teeth 11 and 21, and elastic traction was initiated to implement the orthodontic correction process. This appliance design exemplifies a targeted and controlled approach to achieve optimal tooth movement in the desired direction during the repositioning process. After eight weeks (six months post-trauma) of continuous elastic traction, both maxillary central incisors erupted in the oral cavity, and both were mesiolingually rotated. Hence, to derotate, it was decided to do sectional bonding of maxillary central incisors. Gingivectomy was performed with electrocautery to expose more coronal tissue of 11 and 21 to facilitate bonding (Figure 2).

**Figure 3:**
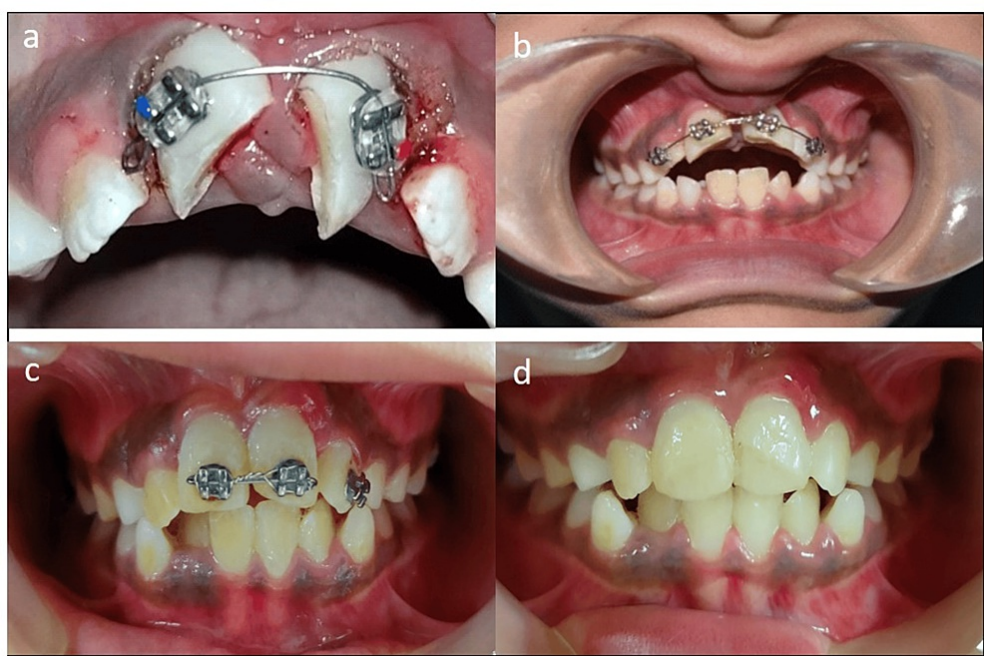
a) Brackets bonded on teeth 11 and 21, b) sectional bonding for proper alignment of incisors and REP initiated, c) reviewed after two months of REP, d) 20 months follow-up after initial intervention (nine months after REP). REP - Regenerative endodontic procedures

Brackets were placed, and after two weeks, most of the rotational correction was achieved. However, as the maxillary incisors derotated, an open bite was observed, so maxillary lateral incisors were also bonded for further tooth movement and proper alignment. To avoid the development of adaptive tongue thrust, the patient was advised on tongue swallowing exercises. Following a negative response to the pulp sensibility test in both maxillary central incisors, indicative of non-vitality, endodontic treatment was initiated due to periapical radiolucency and external inflammatory root resorption (ERR). A regenerative endodontic procedure (REP) was planned for teeth 11 and 21, characterized by an open apex, bulbous canal, and thin dentinal walls. During the first visit, access opening, working length determination, and initial apical filing were conducted with minimal instrumentation. Canal irrigation using 1.5% sodium hypochlorite (20 mL/canal) was performed, and calcium hydroxide was placed as an intracanal agent to arrest ERR. After two weeks, the tooth exhibited asymptomatic behavior and lacked sensitivity to percussion (Figure 3).

**Figure 4:**
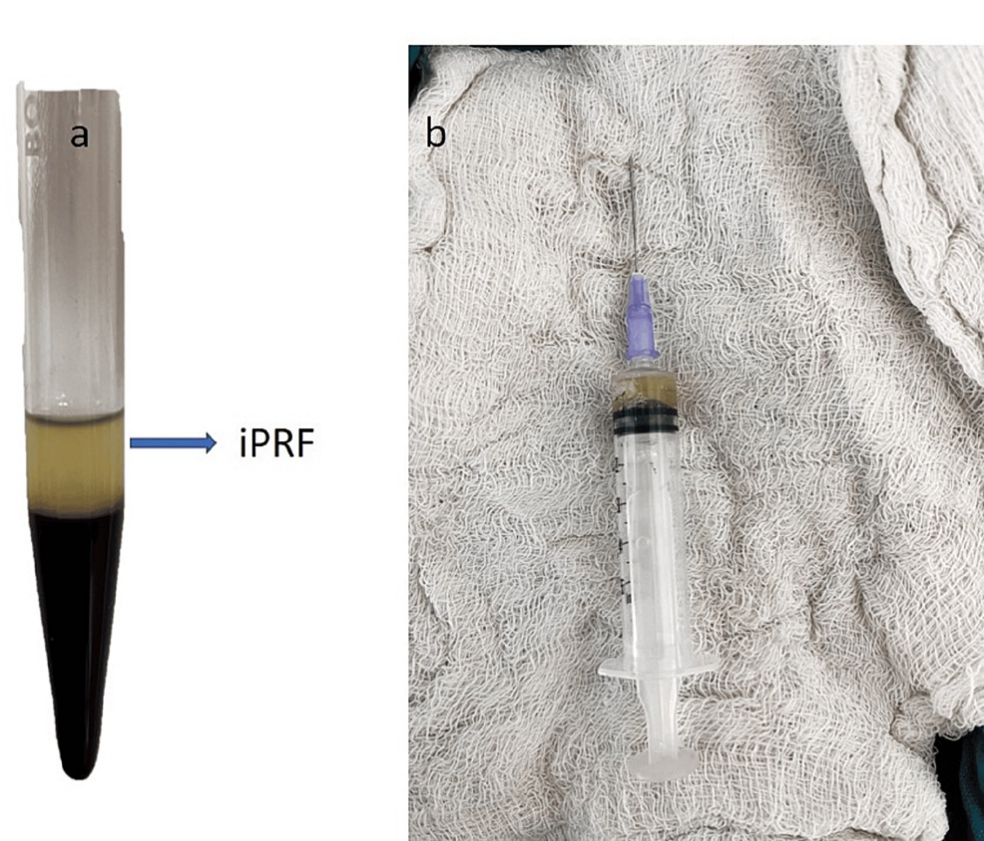
a) iPRF centrifuged at 800 rpm for 3 min and b) iPRF collected in a disposable syringe. iPRF - Injectable platelet-rich fibrin

For REP, injectable platelet-rich fibrin (i-PRF) was obtained by centrifuging 10 mL of whole blood, and the upper portion containing i-PRF was collected with a disposable syringe after centrifugation (Figure 4). Canals were irrigated with 17% EDTA under local anesthesia without adrenaline, followed by drying with sterile paper points. i-PRF was placed into the canals of teeth 11 and 21. A scaffold was created using white mineral trioxide aggregate (MTA) just below the coronal chamber, covered with 3-4 mm of GC Fuji IX glass ionomer cement (GC Corporation, Japan) as interim restoration, later replaced by composite restoration.

**Figure 5:**
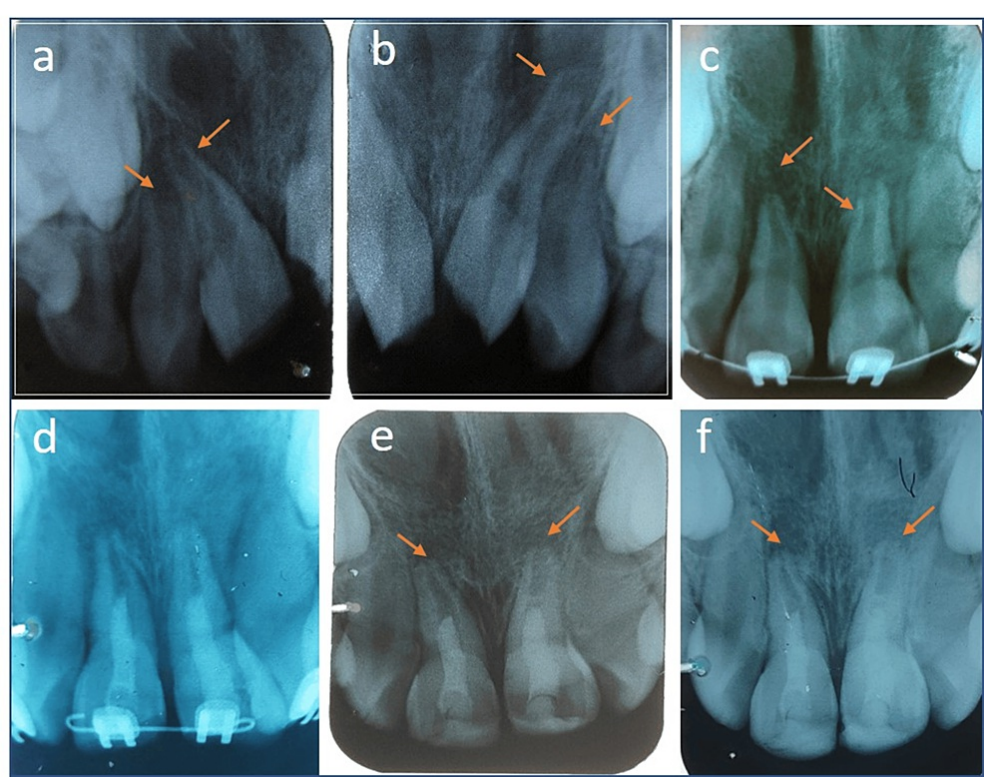
a, b) Intruded teeth 11, 12, 21, 22 with open apices c) after eight months post-trauma showing periapical radiolucency and open apex, bulbous canals, thin root walls. d) REP done in 11 and 21. e) Root end closure observed in 11 and 21. f) 20 months follow-up after intervention (nine months after REP).

**Figure 6:**
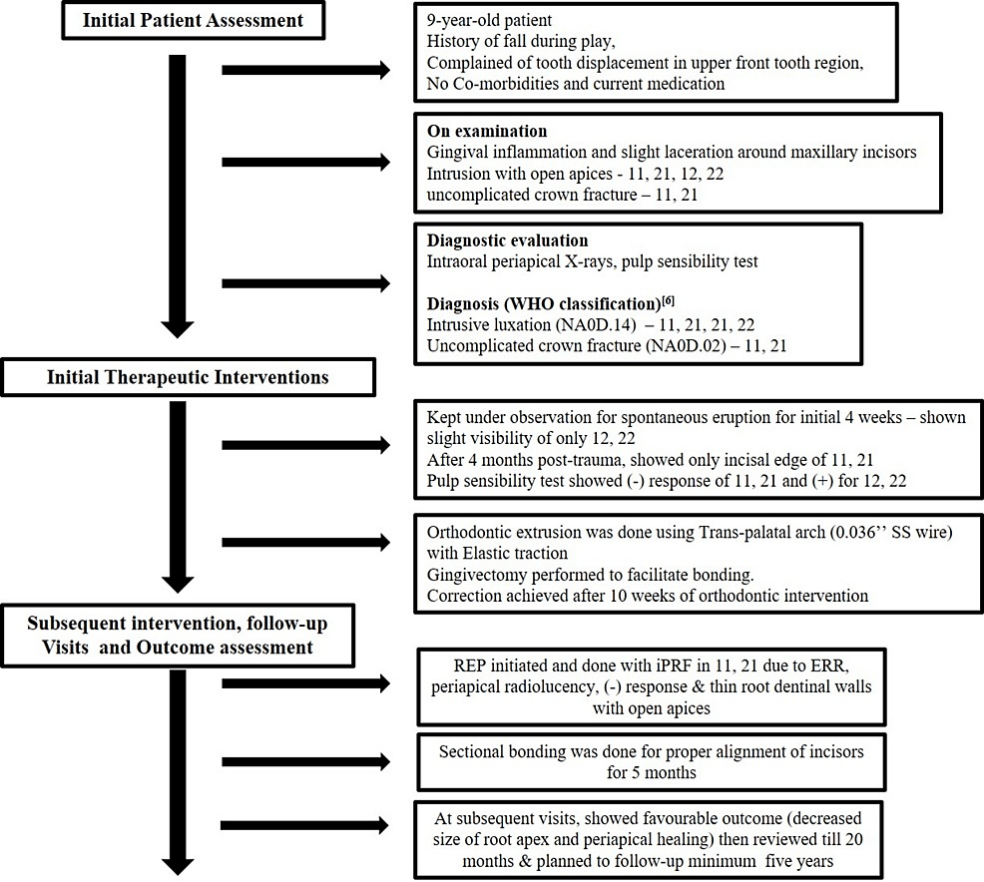
Flow chart depicts the case description as per CARE guidelines. Ref [6]^ ^followed the WHO classification.

Five months following the sectional bonding, marking 11 months post injury, teeth 11, 21, 12, and 22 demonstrated successful alignment, and the open bite had notably reduced. Subsequently, the patient underwent a review for 20 months after the initial intervention (nine months after REP) (Figure 3). The plan for future orthodontic treatment will be devised once all permanent teeth have erupted. The radiographic images were shown (Figure 5). Despite the positive outcomes, an extended follow-up is essential to monitor any potential adverse events, such as ankylosis and pulp necrosis. The case report was summarized in the flow chart (Figure 6).

## Discussion

The management of traumatic intrusion in young permanent incisors can be a complex and challenging process, often requiring careful consideration of a range of factors to achieve predictable, long-term outcomes. Shalish et al. found that intrusive luxation predominantly affects maxillary central incisors and that 90% of affected teeth had already lost their vitality before receiving orthodontic treatment [2]. Our case report findings were consistent with this observation. The severity of intrusion and the stage of root development are two of the most important factors that must be taken into account in determining the appropriate treatment protocol. Depending on the specific circumstances, treatment options may include passive re-eruption, orthodontic intervention, or surgical repositioning. When dealing with immature teeth, it is often preferable to wait and observe the tooth's progress, as they have the potential for spontaneous re-eruption and repair [5]. In the case report, orthodontic repositioning was planned to prevent possible ankylosis. In cases where orthodontic repositioning is deemed necessary, the use of elastic traction has been shown to produce better outcomes compared to surgical intervention, which can be less favorable [7-9]. It has been reported that orthodontic appliances can carry potential risks, such as unintended tooth movement, gingival recession, root resorption, and pulp necrosis. However, in our case report, we have managed to reduce these risks by performing targeted and controlled orthodontic movement while scheduling regular follow-up visits. We have informed the parents about the importance of these follow-ups. Teeth with both traumatic intrusion and orthodontic repositioning have lower survival rates than those with only orthodontic extrusion [2].

However, in cases of severe intrusion, endodontic challenges may arise due to limited accessibility, which highlights the importance of a multidisciplinary approach. It is often necessary to employ a combination of techniques to achieve optimal results. Liang et al. [10] and Nageh et al. [11] conducted a study on the use of i-PRF as a novel biological scaffold for REP. Our case report used a similar treatment approach, and, to our knowledge, it is the only case report that has used i-PRF in immature teeth. This approach is designed to promote continuous root length thickening and maintain the patient's asymptomatic status. The technique is time-consuming and sensitive. There are insufficient clinical trials to evaluate potential complications.

After a five-month initial observation period, this technique was employed in a patient, and a 20-month review showed favorable outcomes, including asymptomatic and periapical healing, and increased root wall thickness. This achieved the successful primary and secondary goals of REP. The patient's compliance with yearly clinical and radiographic evaluations for at least five years is necessary to assess the long-term efficacy and adverse outcomes of treatment [5,12].

## Conclusions

This case report highlights the successful interdisciplinary management of traumatic intrusion and rotation in immature upper permanent incisors. The initial observation, followed by targeted orthodontic intervention and RET using i-PRF, resulted in positive outcomes after 20 months. The case report underscores the challenges in treating traumatic dental injuries in children and emphasizes the importance of tailored comprehensive approaches. While the 20-month follow-up showcases successful outcomes, extended monitoring remains imperative to assess long-term efficacy and identify potential complications. This case report contributes valuable insights into the evolving strategies in pediatric traumatic dental injury management.

## References

[1] Hashim R, Alhammadi H, Varma S, Luke A (2022). Traumatic dental injuries among 12-year-old schoolchildren in the United Arab Emirates. Int J Environ Res Public Health.

[2] Shalish M, Abed J, Keinan D, Slutzky-Goldberg I (2024). The consequences of orthodontic extrusion on previously intruded permanent incisors-a retrospective study. Dent Traumatol.

[3] Andreasen JO, Bakland LK, Matras RC, Andreasen FM (2006). Traumatic intrusion of permanent teeth. Part 1. An epidemiological study of 216 intruded permanent teeth. Dent Traumatol.

[4] Krastl G, Weiger R, Filippi A (2021). Endodontic management of traumatized permanent teeth: a comprehensive review. Int Endod J.

[5] Bourguignon C, Cohenca N, Lauridsen E (2020). International Association of Dental traumatology guidelines for the management of traumatic dental injuries: 1. fractures and luxations. Dent Traumatol.

[6] Petti S, Andreasen JO, Glendor U, Andersson L (2022). Na0d - the new traumatic dental injury classification of the World Health Organization. Dent Traumatol.

[7] Şermet Elbay Ü, Elbay M, Kaya E, Sinanoglu A (2015). Management of an intruded tooth and adjacent tooth showing external resorption as a late complication of dental injury: three-year follow-up. Case Rep Dent.

[8] Chaushu S, Shapira J, Heling I, Becker A (2004). Emergency orthodontic treatment after the traumatic intrusive luxation of maxillary incisors. Am J Orthod Dentofacial Orthop.

[9] Andreasen JO, Bakland LK, Andreasen FM (2006). Traumatic intrusion of permanent teeth. Part 3. A clinical study of the effect of treatment variables such as treatment delay, method of repositioning, type of splint, length of splinting and antibiotics on 140 teeth. Dent Traumatol.

[10] Liang Y, Ma R, Chen L (2021). Efficacy of i-PRF in regenerative endodontics therapy for mature permanent teeth with pulp necrosis: study protocol for a multicentre randomised controlled trial. Trials.

[11] Nageh M, Ibrahim LA, AbuNaeem FM, Salam E (2022). Management of internal inflammatory root resorption using injectable platelet-rich fibrin revascularization technique: a clinical study with cone-beam computed tomography evaluation. Clin Oral Investig.

[12] Kim SG, Malek M, Sigurdsson A, Lin LM, Kahler B (2018). Regenerative endodontics: a comprehensive review. Int Endod J.

